# Diagnostic Stability and Phenotypic Differences Among School-Age Children Diagnosed With ASD Before Age 2

**DOI:** 10.3389/fpsyt.2022.805686

**Published:** 2022-03-15

**Authors:** Rebecca J. Landa, Rachel Reetzke, Calliope B. Holingue, Dana Herman, Christine Reiner Hess

**Affiliations:** ^1^Center for Autism and Related Disorders, Kennedy Krieger Institute, Baltimore, MD, United States; ^2^Department of Psychiatry and Behavioral Sciences, The Johns Hopkins University School of Medicine, Baltimore, MD, United States; ^3^Department of Mental Health, Johns Hopkins Bloomberg School of Public Health, Baltimore, MD, United States

**Keywords:** autism spectrum disorder, infant siblings, elevated likelihood, clinical diagnosis, diagnostic stability, developmental trajectories, predictors, late diagnosis

## Abstract

Given the importance of early detection, it is critical to understand the non-linearity in manifestation of ASD before age 24 months, when ASD symptoms are beginning to consolidate, through the age of 36 months when stability of ASD diagnosis is reportedly high into school-age when increased demands may challenge previously successful compensatory processes and permit first ASD detection. We employed a prospective, longitudinal design focused on children with an older sibling with ASD (*n* = 210) who received diagnostic evaluations at mean ages of 15.4 months (Time 1), 36.6 months (Time 2), and 5.7 years (Time 3) to examine: (1) diagnostic stability, (2) developmental trajectories associated with different patterns of ASD vs. non-ASD classifications, and (3) predictors of classification group over time. Clinical best estimate (CBE) diagnosis of ASD or non-ASD was made at each time point. Linear mixed-effects models were implemented to examine differences in developmental trajectories of stable and dynamic diagnostic groups. Multinomial logistic regression analyses were used to examine predictors of the likelihood of belonging to each CBE diagnostic classification group. Results revealed that sensitivity and stability of an ASD diagnosis significantly increased from Time 1 (sensitivity: 52%; stability: 63%) to Time 2 (sensitivity: 86%; stability: 68%). Different developmental trajectories of autism symptom severity and non-verbal and verbal IQ were observed across groups, with differences first observed at Time 1 and becoming more pronounced through Time 3. Presence of restricted and repetitive behaviors as well as limitations in initiation of joint attention and expressive language skills differentially predicted the likelihood of belonging to the different CBE diagnostic classification groups. Results suggest that ASD symptoms may emerge or attenuate over time, with some children meeting diagnosis at follow-up, and other children no longer meeting diagnostic criteria. From a systems perspective, diagnostic non-linearity may be viewed as a dynamic developmental process, where emergent properties arising from various biological, genetic, and experiential factors interact, culminating in phenotypic phenomena that change over time. Clinical implications include extending universal ASD and social communication screening into school-age, supporting families' understanding of diagnostic shifts, and ensuring unbiased diagnostic decision-making when following children with ASD.

## Introduction

Autism spectrum disorder (ASD) affects multiple developmental systems, presents along a continuum of severity (i.e., in social communication, language, and cognitive functioning, as well as in restricted and repetitive behaviors) (1, 2) and often, co-occurs with other conditions (3, 4). These factors, along with the considerable heterogeneity of ASD, may contribute to complexities in attaining early and accurate diagnosis. Indeed, families often describe the experience of obtaining an ASD diagnosis as a ‘diagnostic odyssey' (5). The process of obtaining diagnostic assessments and coming to diagnostic clarity often is lengthy and families may encounter diagnostic disagreement between professionals at a given time, and across time (6–8). To better understand diagnostic stability, developmental trajectories associated with different patterns of ASD vs. non-ASD classifications over time, and predictors of those patterns, we employed a prospective, longitudinal design and focused on children with and without elevated likelihood for ASD (ELA) from mean age 15 months to school-age.

Much research has been focused on early detection of ASD to support families' ability to access intervention during a period of robust neuroplasticity (9, 10). Currently, ASD diagnosis can be made prior to 24 months by well-trained experts (7, 11, 12). Considerable efforts have been made to elucidate whether diagnostic stability is achieved when ASD diagnosis is made within a particular age range. Many of such studies have focused on follow-up of young children referred for clinical ASD assessment or children who received community-based ASD screening; most used *Diagnostic and Statistical Manual of Mental Disorders*, Fourth *Edition* (DSM-IV) (13) or Fourth *Edition, Text Revision* (DSM-IV-TR) (14) diagnostic categories. While sample sizes were small in most studies, high stability rates (72–100%) have been reported when first assessments occurred near the second birthday and follow-up assessments occurred from about 1–7 years later (1, 15–21). In a study of 172 2-year-olds referred for clinical assessment to rule out autism and followed to age 9 years, Lord et al. (1) reported the highest ASD diagnostic stability rates for children diagnosed with autism, as opposed to Pervasive Developmental Disorder-Not Otherwise Specified (PDD-NOS), at 24 months (using DSM-IV criteria). Most shifts in diagnosis were from PDD-NOS to autism, indicating greater symptom expression over time. Despite overall high stability rates across the autism spectrum, 10% of the sample received their first ASD diagnosis at age 5 or 9 years (1) and 10% of the original 2-year-old autistic children followed to age 19 years had neurotypical presentations (22). Following 123 children from Lord et al.'s (1) original sample into adulthood, Pickles et al. (23) found that adult outcomes were well-predicted by autism symptom severity and IQ beginning at age 2 years, with increasing predictive strength between ages 2 and 9 years.

Three recent studies examined diagnostic stability in ASD screen-positive children. The first diagnostic assessment in these studies was conducted between age 12 months and median age 27.7 months, with follow-up assessment at ages 3– 4 years; all used DSM-5 criteria (24–26). ASD diagnostic stability in these three studies (83.3 to 88.3%) replicated prior reports by others, indicating that diagnostic criteria (i.e., DSM-IV, DSM-IV-R, DSM-5) is not a driving factor in stability rate. Furthermore, high stability was identified in an economically and ethnically/racially diverse sample (25). Giserman-Kiss and Carter (25) found that age at first diagnosis was a strong predictor of unstable ASD diagnosis, with increasing age at first diagnosis being more strongly related to losing the ASD diagnosis at the follow-up assessment (25).

Another approach to elucidating ASD diagnostic stability involves use of a longitudinal prospective design, where children at elevated familial likelihood of developing ASD (ELA; younger siblings of a child with ASD) are followed from infancy. About one in five children with ELA will receive an ASD diagnosis by age 3 years (27, 28). Advantages of this approach include sample ascertainment independently of a clinical diagnosis or screening status (at time of recruitment), careful phenotypic assessment by experts in very early ASD diagnosis, use of state-of-the-science diagnostic procedures at established ages, and the ability to follow children who demonstrate a continuum of developmental functioning and range of diagnostic classifications (e.g., neurotypical, language disorder, ASD). This approach affords the ability to examine dynamic developmental processes, elucidate stability of ASD diagnosis and its relation to age of diagnosis, identify trajectories of dimensional characteristics of development, and identify predictors of different ASD positive/negative classifications over time.

Two large-sample (*n* ≥ 381) prospective longitudinal studies of ELA children focused on stability of early ASD diagnosis (7, 12). In both, age at first ASD vs. non-ASD classification was determined at 18 months, with diagnostic confirmatory assessment at age 36 months. Ozonoff et al. (7) reported diagnostic stability (positive predictive value) decreased from 93% to 82% for diagnoses given at ages 18 and 24 months, respectively. Negative predictive value increased with age from 82 to 87% at the 18- and 24-month assessments, respectively (7). In contrast to Ozonoff et al. (7), Zwaigenbaum et al. (12) found that stability of an ASD diagnosis increased from age 18 (83%) to 24 months (92%). However, in line with Ozonoff et al. (7), this study found that negative predictive value increased with age from 75% at 18 months, to 85% at 24 months.

Longer-term ASD diagnostic stability has been examined in two prospective longitudinal studies of ELA children. In Brian et al.'s (29) study, 17 of 18 ELA children diagnosed with ASD at age 3, retained their ASD diagnosis at age 9.5 years. Stability of ASD diagnosis was 94.4%, and stability of non-ASD classification was 87.8%. Six of the 49 children classified as non-ASD at age 3, received a later diagnosis of ASD at 9.5 years (12.2%, false negative), while one child diagnosed with ASD lost the diagnosis at 9.5 years (1/18 = 5% false positive). The later-diagnosed children had higher receptive language and lower ASD symptoms at age 3 than those diagnosed with ASD at age 3. In another prospective study, 67 children with ELA were followed from age 3 to age 7 years (30). Stability for ASD and non-ASD classification was 76.9 and 82.8%, respectively. Five of the children classified as non-ASD at age 3 were later diagnosed with ASD at age 7 years (17.2%, false negative). Twenty-three percent of children diagnosed with ASD at age 3 lost their ASD diagnosis by age 7 (false positive). Children with ASD in these two studies had Full Scale IQs in the average range and Autism Diagnostic Observation Schedule-Generic (ADOS) Comparison Severity Scores (31) of 7.0 (on a 10-point scale, with 10 being the most severe) at age 9.5 years (29) and of 6.6 at age 7 years (30). Thus, neither ASD symptom severity nor cognitive functioning explain the discrepancy in stability across these two studies.

In an attempt to elucidate later diagnosed cases of ASD, Ozonoff et al. (32) closely examined assessment data on 14 children with ELA who, despite having had repeated, detailed clinical phenotyping assessments by ASD experts at earlier ages, were not diagnosed with ASD until between 5 and 7 years of age. Overall, these children exhibited significantly higher levels of cognitive functioning and less autism symptomatology compared to the early-diagnosed children. However, 36-month data revealed heterogeneity in these later-diagnosed children. At age 3 years, nine children had been classified as typically developing (TD) and the remaining five had been classified as non-ASD and also non-TD, but no other diagnosis was given. Ozonoff et al.'s (32) findings extend, over a wider age range, Landa et al.'s (33) finding of increasing departure from neurotypical behavior over time in children later diagnosed with ASD, and Landa et al.'s (11) finding of heterogeneous developmental trajectories in children with later-diagnosed ASD.

Reports of diagnostic instability (e.g., “false positives” or “lost diagnosis”) and “missed” cases (“false negatives” or “later diagnosed”) demonstrate the variability and non-linearity of the ASD phenotype in early development. This non-linearity has historically been attributed to failure to recognize early signs of ASD, wait-and-see perspectives, overshadowing of ASD symptoms by other conditions at a younger age, differences in professionals' thresholds for diagnosing ASD (34), or, relatedly, clinical error. Ozonoff et al.'s (32) data contradict all of these explanations except perhaps clinical error. We propose that a developmental explanation for non-linearity in ASD diagnosis over time also should be considered. For example, there is a prodromal period for ASD, with symptom consolidation usually occurring in the second and third years of life. This could explain why very young children may be diagnosed with ASD later in development, despite not receiving an ASD diagnosis initially at ages 12 to 18 months (7, 12, 26, 33). Furthermore, longer-term follow-up studies (1, 22, 28, 29, 31) demonstrate that ASD symptoms may worsen, emerge, or attenuate over time, with some children meeting ASD diagnosis at follow-up, and other children no longer meeting diagnostic criteria. A systems, rather than reductionist, perspective is needed to interpret these discrepancies in phenotypic presentation over time (35–37). From a systems perspective, diagnostic and developmental non-linearity may be viewed as a dynamic developmental process, where emergent properties arising from various factors (experiences, cognitive propensities, biology, etc.) interact, culminating in certain developmental phenomena that change over time (38).

The present study has three aims. First, we examine, for the first time in a prospective, longitudinal sample of children with ELA, non-linearity in manifestation of ASD from mean age 15 months (Time 1), when ASD symptoms are beginning to consolidate, to the age of 36 months when stability of ASD diagnosis is reportedly high for the long term (Time 2), and at early school-age (mean age 5.7 years) (Time 3). Second, we take a dimensional approach to examine phenotypic differences in developmental trajectories among stable ASD and non-ASD groups as well as diagnostically dynamic groups defined by shifts in non-ASD/ASD status. Finally, we examine early and intermediate predictors of those groupings. We chose expressive language, initiation of joint attention, and restricted and repetitive behaviors (RRBs) as predictors given that low levels of expressive language and initiation of joint attention, and presence of RRBs are known to be characteristic of ASD in early development (33, 39, 40). Further, early expressive language and frequency of initiation of joint attention are predictive of pragmatic communication skills (a core feature of ASD) at adolescence (33, 39, 41).

## Materials and Methods

### Participants

Participants were recruited for a prospective, longitudinal study of ASD. This study was approved by the Johns Hopkins Medicine Institutional Review Board. Caregivers signed written informed consent prior to their child's participation in this study. All procedures were carried out following the approved guidelines.

Two-hundred and ten siblings of a proband with ASD (hereafter, ELA) were drawn from a larger prospective, longitudinal study. Inclusion criteria for our analytic sample consisted of children who had complete data at three time points: Time 1 (range = 12.4–25.4 months, M_age_ = 15.4 months, SD_age_ = 2.6 months), Time 2 (range = 29.98–41.98, months, M_age_ = 36.6 months, SD_age_ = 1.9 months), and Time 3 (range = 48.0–104.7 months, M_age_ = 68.4 months, SD_age_ = 12.6 months). Exclusion criteria consisted of non-primary English language speakers (language measures are normed on English speakers), birth weight <1,500 grams, severe birth trauma, severe birth defects, head injury, and prenatal illicit drug or alcohol exposure.

At each assessment time point, participants received a battery of standardized assessments, including a measure of verbal and non-verbal intelligence and the Autism Diagnostic Observation Schedule, Generic (ADOS-G) (42) or the Autism Diagnostic Observation Schedule, Second Edition (ADOS-2) (31). Clinical judgment of ASD status (ASD or non-ASD) was made by an expert clinical researcher conducting the child's assessment based on the child's assessment data (including both examiner-administered and parent-report measures), and direct observation of the child's behavior during the evaluation session (described further below).

### Measures

#### Clinical Best Estimate (CBE) Diagnosis

CBE diagnosis at each time point (Time 1, Time 2, Time 3) was made by a research-reliable, clinical research examiner with a master's or doctoral degree and expertise in early diagnosis of ASD. All examiners were blind to risk group membership (the larger study also included children without ELA) and to diagnoses made at earlier time points. CBE of ASD was made based on: (1) ADOS administration, (2) direct observation of the child during assessment visits, (3) parent report on child developmental history, and (4) DSM-IV (13) or DSM-5 (43) criteria for ASD. In line with previous studies examining stability of ASD diagnosis over time (7, 12, 28, 29), all clinical diagnoses were dichotomized as ASD or non-ASD for further analysis.

#### Autism Diagnostic Observation Schedule

The Autism Diagnostic Observation Schedule (ADOS) (31, 42) is a standardized, semi-structured, play-based clinician-administered measure designed to assess ASD symptomatology related to communication, social interaction, play and restricted, repetitive behaviors and interests (31). The ADOS consists of different modules, with module selection based on chronological age and language ability at time of testing. In the current study, the ADOS was administered by research-reliable staff as part of a comprehensive clinical research evaluation. During the Time 1 assessment, children completed the ADOS-2 Toddler Module or ADOS-G Module 1 (minimal to no language) depending on when they entered the study (before or after publication of the ADOS-2). At the Time 2 assessment, children completed the ADOS-G or ADOS-2 Module 1 or 2 (non-echoed phrase speech). At the Time 3 assessment, children completed the ADOS-G or ADOS-2 Module 1, 2 (non-echoed phrase speech), or 3 (fluent language). Across all modules, an ADOS Calibrated Severity Score (CSS; possible range 1 to 10) was derived, which reflects the relative severity of autism-specific symptoms and allows comparisons of the same child over time, comparisons across modules, and comparisons across ADOS-G and ADOS-2 versions. The Toddler Module CSS reported in the current study was calculated based on Esler et al. (44). Higher ADOS scores and CSS reflect greater ASD symptom severity. The CSS was used for group comparisons at all three time points.

Since joint attention limitations are often observed in young children with ASD (45) and joint attention skills strongly predict early language development (46) as well as later pragmatic communication skills (41), we examined Time 1 and Time 2 initiation of joint attention (IJA) behavior as predictors of Time 3 diagnostic classification. An IJA composite variable was derived using the sum of ADOS ratings from three items: *Initiation of Joint Attention, Showing*, and *Giving* (47). To account for differences across the ADOS Toddler Module and Module 1 in the range of item ratings and the operational definitions of those ratings, recoding of items was necessary to align item ratings across modules. For the *Initiation of Joint Attention* and *Showing* items, we recoded such that ratings of 0 = 0 and ratings of 1, 2, and 3 were recoded to “2.” In terms of the *Giving* item, since the ratings were nearly identical across the modules we maintained 0 = 0, 1 = 1, and 2 = 2 for the Toddler Module and Module 1, but recoded Module 2 ratings of 3 to 2 (there is no rating of “3” on Module 1).

Since restricted and repetitive behaviors are core features of ASD (43) and an early behavioral symptom of ASD (40), we examined Time 1 and Time 2 ADOS restricted and repetitive behaviors (RRB) domain score as a predictor of Time 3 diagnostic classification. The RRB domain score was specific to each module algorithm and consisted of the sum of ADOS ratings from a unique combination of the following items: *Intonation of Vocalizations or Verbalizations* (Toddler Module and Module 1); *Stereotyped/Idiosyncratic Use of Words or Phrases* (Modules 1–3); *Unusual Sensory Interest in Play Material/Person* (Toddler Module and Modules 1–3), *Hand and Finger Movements/Posturing* (Toddler Module); *Hand and Finger and Other Complex Mannerisms* (Modules 1–3), *Unusually Repetitive Interests or Stereotyped Behaviors* (Toddler Module and Modules 1–2), and *Excessive Interest in Unusual or Highly Specific Topics/Objects or Repetitive Behaviors* (Module 3).

#### Mullen Scales of Early Learning

The Mullen Scales of Early Learning (MSEL) (48) is a standardized, norm-referenced developmental test for ages birth to 68 months. Four subscales were administered to assess children's development at Time 1 and Time 2: Fine Motor, Receptive Language, Expressive Language, and Visual Reception. Visual Reception measures non-verbal cognitive skills including visual processing, visual spatial, memory, and problem solving skills (49). Verbal and non-verbal developmental quotients (DQs) were calculated by dividing each MSEL subscale age-equivalent score by the child's chronological age and multiplying by 100 (50). Verbal (i.e., the average of the Receptive and Expressive Language DQs) and non-verbal (i.e., the average of the Fine Motor and Visual Reception DQs) DQs were derived to estimate Time 1 and Time 2 cognitive functioning to allow for comparison with the Stanford-Binet Intelligence Scales, Fifth Edition which was administered at Time 3. For ease of reference, DQ is referred to as IQ herein. The Expressive Language T Score was calculated to examine expressive language at Time 1 and Time 2, as a predictor of Time 3 diagnostic classification.

#### Stanford-Binet Intelligence Scales, Fifth Edition

The Stanford-Binet Intelligence Scales, Fifth Edition (SB-5) (51) is a standardized, norm-referenced measure of five cognitive factors of intelligence for ages 2–85 years: Fluid Reasoning, Knowledge, Quantitative Reasoning, Visual-Spatial Processing, and Working Memory. Each factor has a verbal and non-verbal domain resulting in 10 index scores. Index scores convert to composite standard scores for Verbal IQ, Non-verbal IQ, and Full Scale IQ (*M* = 100, *SD* = 15). Verbal IQ and Non-verbal IQ were used to estimate Time 3 cognitive functioning.

### Statistical Analysis

Descriptive data were analyzed for four school-age outcome groups (Stable ASD, Lost Diagnosis, Later Diagnosed, and Stable non-ASD) to describe demographic characteristics (see Table 1). Given previous findings suggesting potential female resilience in ASD (47, 52), Fisher's exact tests were used to examine whether sex distributions significantly differed by diagnostic group.

**Table 1:** Participant characteristics.

	**Later diagnosed**	**Lost diagnosis**	**Stable ASD**	**Stable non-ASD**
	**(*N* = 23)**	**(*N* = 23)**	**(*N* = 19)**	**(*N* = 145)**
Age at Time 1	14.37 (0.55)	15.59 (2.77)	15.70 (2.85)	15.44 (2.68)
Age at Time 2	36.19 (1.32)	35.74 (2.56)	36.18 (2.27)	36.83 (1.76)
Age at Time 3	68.94 (15.75)	70.45 (11.52)	73.63 (12.49)	67.24 (12.07)
Sex				
F	3 (13.0%)	7 (30.4%)	3 (15.8%)	70 (48.3%)
M	20 (87.0%)	16 (69.6%)	16 (84.2%)	75 (51.7%)
Race				
Asian	1 (4.3%)	0 (0.0%)	0 (0.0%)	1 (0.7%)
Black	0 (0.0%)	0 (0.0%)	0 (0.0%)	3 (2.1%)
Hispanic	0 (0.0%)	0 (0.0%)	0 (0.0%)	1 (0.7%)
Multiracial	3 (13.0%)	3 (13.0%)	5 (26.3%)	4 (2.8%)
White	11 (47.8%)	17 (73.9%)	12 (63.2%)	99 (68.3%)
Unknown/not reported	8 (34.8%)	3 (13.0%)	2 (10.5%)	37 (25.5%)
Maternal education				
Associates degree	2 (8.7%)	0 (0.0%)	2 (10.5%)	2 (1.4%)
College degree or higher	18 (78.3%)	19 (82.6%)	12 (63.2%)	120 (82.8%)
High school or vocational training	3 (13.0%)	3 (13.0%)	4 (21.1%)	14 (9.7%)
Unknown/not reported	0 (0.0%)	1 (4.3%)	1 (5.3%)	9 (6.2%)
Household income				
$60 k or less	4 (17.4%)	1 (4.3%)	2 (10.5%)	5 (3.4%)
$61 k or higher	9 (39.1%)	3 (13.0%)	6 (31.6%)	55 (37.9%)
Unknown/not reported	10 (43.5%)	19 (82.6%)	11 (57.9%)	85 (58.6%)

*ASD, autism spectrum disorder; F, female; M, male*.

To examine the stability at early school-age (mean age 5.7 years) of ASD CBE diagnostic classifications made at the mean ages of 15 (Time 1) and 36-months (Time 2), we examined sensitivity, specificity, positive predictive value (PPV), and negative predictive value (NPV). Aligned with Ozonoff et al. (7), differences in sensitivity and specificity for Time 1 and Time 2 CBE diagnostic classification were tested using McNemar's test.

Linear mixed-effects models were implemented to examine differences in longitudinal trajectories of autism symptom severity, as well as verbal and non-verbal IQ among participants with stable vs. dynamic ASD diagnoses. For each dependent variable, we fit a model that included fixed effects for outcome group (Stable ASD, Later Diagnosed, Lost Diagnosis, with the Stable non-ASD group as reference) and time point (Time 2, Time 3, with Time 1 as the reference), and time point by outcome group interactions. Participant intercept was included as a random effect to account for correlation due to repeated measurements.

Finally, we used multinomial logistic regression analyses to examine predictors of the likelihood of belonging to each Time 3 outcome group (i.e., Later Diagnosed, Lost Diagnosis, Stable ASD, with Stable non-ASD, as reference). Predictor variables of interest included: the MSEL Expressive Language T Score, an IJA derived composite score from the ADOS, as well as the Restricted and Repetitive Behaviors (RRB) domain score from the ADOS.

For all statistical analyses, an alpha of *p* < 0.05 was considered statistically significant and 95% confidence intervals were estimated. All analyses were carried out in R Studio (Version 1.4.1106; R Version 4.0.4, RStudio Team, 2021) using the “epiR” (Version 2.0.36), “lme4” (Version 1.1-26), “lmerTest” (Version 3.1-3), and “nnet” (Version 7.3-16) packages.

## Results

### Diagnostic Stability

At mean ages 15.4 months (Time 1), 36.6 months (Time 2), and 5.7 years (Time 3), 35, 53, and 42 children received an ASD diagnosis, respectively. In line with previous investigations (7, 12), eight patterns of CBE diagnostic stability were derived based on an outcome of ASD vs. non-ASD at each time point. As shown in Table 2, some children presented with stable CBE diagnostic classifications across all three time points (e.g., AAA, NNN; where “A” = ASD, and “N” = non-ASD). Other children exhibited a dynamic pattern of CBE diagnostic classification over time (e.g., AAN, ANA, NAN). To examine phenotypic differences in developmental trajectories among these groups, we derived four stability groups based on the eight classification patterns (7), with Time 3 CBE diagnostic classification as the outcome standard. A Stable ASD group (*n* = 19) was defined as meeting criteria for ASD across all time points (e.g., AAA) and a Stable non-ASD group (*n* = 145) was defined as not meeting criteria for ASD across all time points (e.g., NNN). Dynamic patterns of diagnostic classifications over time were classified into one of two groups based on CBE diagnostic classification at either Time 1 or Time 2 that differed from Time 3 CBE diagnostic classification. Specifically, a Lost Diagnosis group (*n* = 23) met ASD criteria at Time 1 and/or at Time 2, but not at Time 3, while participants grouped as Later Diagnosed (*n* = 23) did not meet ASD criteria at Time 1 and/or at Time 2, but did meet criteria at Time 3 (see Table 2 for summary). Compared to the Stable non-ASD group, the Later Diagnosed, Lost Diagnosis, and Stable ASD groups consisted of a greater proportion of males relative to females (all *p*s ≤ 0.01). Compared to the Lost Diagnosis group, the Later Diagnosed group consisted of a greater proportion of males, with a significantly greater proportion of females observed in the Lost Diagnosis group (30%) compared to the Later Diagnosed group (13%) (*p* = 0.03). No other significant group differences in sex distributions were observed (all *p*s ≥ 0.07).

**Table 2:** Patterns of Clinical Best Estimate outcome classifications by time point.

**Clinical best estimate outcome**			**Total**	**ASD at school-age**	**Non-ASD at school-age**	**Classification**
			**(*n* = 210)**	**(*n* =4 2)**	**(*n* = 168)**	
**Time 1**	**Time 2**	**Time 3**				
A	A	A	19	45%		Stable ASD
A	A	N	7		4%	Lost diagnosis
A	N	N	6		4%	Lost diagnosis
N	A	N	10		6%	Lost diagnosis
A	N	A	3	7%		Later diagnosed
N	A	A	17	40%		Later diagnosed
N	N	A	3	7%		Later diagnosed
N	N	N	145		86%	Stable non-ASD

*ASD, autism spectrum disorder; A, Diagnosis of ASD; N, Classification of non-ASD*.

Psychometric measures at Time 1 and Time 2, with Time 3 as the outcome standard, are presented in Table 3. Sensitivity of ASD diagnosis, established at mean age 5.7 years, significantly increased from Time 1 (52%) to Time 2 (86%) (*p* = 0.01). Indeed, a significant increase in PPV was observed from Time 1 (63%) to Time 2 (68%). This finding may reflect the developmental emergence of the ASD phenotype over time.

**Table 3:** Stability and diagnostic classification parameters at Time 1 and Time 2.

	**ASD at Time 3**		**Non-ASD at Time 3**		**Sensitivity**	**Specificity**	**PPV/stability**	**NPV**
					**(95% CI)**	**(95% CI)**	**(95% CI)**	**(95% CI)**
	**ASD (true positives)**	**Non-ASD (false negatives)**	**ASD (false positives)**	**Non-ASD (true negatives)**				
Time 1 CBE	22	20	13	155	52% (36–68%)	92% (87–96%)	63% (45–79%)	89% (83–93%)
Time 2 CBE	36	6	17	151	86% (71–95%)	90% (84–94%)	68% (54–80%)	96% (92–99%)

*ASD, autism spectrum disorder; CBE, clinical best estimate; CI, confidence interval; PPV, positive predictive value; NPV, negative predictive value*.

### Developmental Trajectories

**Figure 1:**
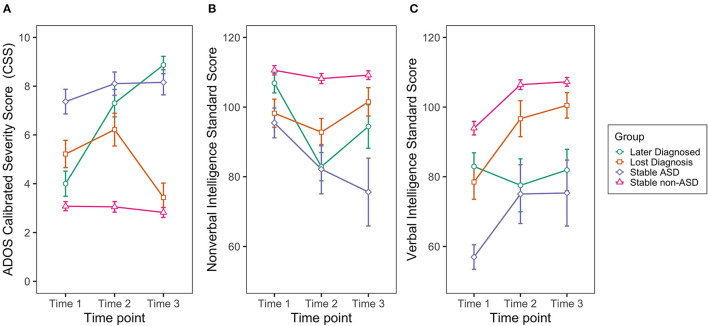
Developmental trajectories of the **(A)** Autism Diagnostic Observation Schedule Calibrated Severity Score (ADOS-CSS); **(B)** Non-verbal and **(C)** Verbal Intelligence (IQ) by group. Non-verbal IQ: Times 1 and 2 = Mullen Scales of Early Learning (MSEL) non-verbal developmental quotient; Time 3 = Stanford-Binet Intelligence Scales, Fifth Edition (SB-5) non-verbal intelligence standard score. Verbal IQ: Times 1 and 2 = MSEL verbal developmental quotient; Time 3 = SB-5 verbal intelligence standard score. Error bars denote ± SEM.

Linear mixed-effects models indicated that the four groups differed in ADOS CSS, non-verbal IQ, and verbal IQ at Time 1. Interactions between outcome group and time were significant for all dependent variables, indicating different group developmental trajectories of ADOS CSS, non-verbal IQ, and verbal IQ from baseline (Time 1) (see Table 4 and Figure 1).

**Table 4:** Parameter Estimates (SE) for linear mixed-effects models examining developmental trajectories of autism symptom severity, as well as non-verbal and verbal IQ.

	**ADOS CSS**		**Non-verbal IQ** ^**a**^		**Verbal IQ** ^**b**^	
	**Estimate (SE)**	***p*-value**	**Estimate (SE)**	***p*-value**	**Estimate (SE)**	***p*-value**
(Intercept)	3.08 (0.17)	<0.001	110.58 (1.2)	<0.001	93.95 (1.44)	<0.001
Later diagnosed	0.92 (0.45)	0.04	−3.69 (3.23)	0.25	−11.00 (3.88)	0.005
Lost diagnosis	2.13 (0.45)	<0.001	−12.34 (3.23)	<0.001	−15.47 (3.88)	<0.001
Stable ASD	4.29 (0.49)	<0.001	−15.12 (3.52)	<0.001	−36.97 (4.22)	<0.001
Time 2	−0.03 (0.22)	0.90	−2.39 (1.42)	0.09	12.48 (1.64)	<0.001
Time 3	−0.26 (0.22)	0.25	−1.47 (1.66)	0.37	12.35 (1.91)	<0.001
Later diagnosed × Time 2	3.33 (0.6)	<0.001	−21.61 (3.85)	<0.001	−17.88 (4.42)	<0.001
Lost diagnosis × Time 2	1.03 (0.6)	0.09	−3.05 (3.85)	0.43	5.71 (4.42)	0.20
Stable ASD × Time 2	0.76 (0.65)	0.24	−11.25 (4.24)	0.01	5.35 (4.87)	0.27
Later diagnosed × Time 3	5.12 (0.6)	<0.001	−10.80 (4.53)	0.02	−13.45 (5.35)	0.01
Lost diagnosis × Time 3	−1.53 (0.6)	0.01	4.76 (4.08)	0.24	8.93 (4.70)	0.06
Stable ASD × Time 3	1.04 (0.65)	0.11	−18.75 (4.75)	<0.001	4.27 (5.61)	0.45

*SE, standard error; ADOS, autism diagnostic observation schedule; CSS, calibrated severity score; IQ, intelligence quotient; ASD, autism spectrum disorder*.

a *Non-verbal IQ: Times 1 and 2 = Mullen Scales of Early Learning (MSEL) non-verbal developmental quotient; Time 3 = Stanford-Binet Intelligence Scales, Fifth Edition (SB-5) non-verbal intelligence standard score*.

b *Verbal IQ: Times 1 and 2 = MSEL verbal developmental quotient; Time 3 = SB-5 verbal intelligence standard score*.

Pairwise comparisons derived from the models (Table 5) indicated that, over time, Later Diagnosed, Lost Diagnosis, and Stable ASD groups had significantly higher ADOS CSS and significantly lower non-verbal and verbal IQ compared to the Stable non-ASD group, with differences first observed at Time 1 and becoming more pronounced through Time 3. At Time 1, however, the Later Diagnosed group did not significantly differ from the Stable non-ASD group in non-verbal IQ; and at Time 3 the Lost Diagnosis group did not differ from the Stable non-ASD group in ADOS CSS.

**Table 5:** Estimated pairwise group differences (SE) in autism symptom severity, non-verbal intelligence, and verbal intelligence across all time points.

	**Later diagnosed**	**Lost vs. stable**	**Stable ASD vs**.	**Later diagnosed**	**Lost diagnosis**	**Later diagnosed**
	**vs. stable non-ASD**	**diagnosis non-ASD**	**stable non-ASD**	**vs. stable ASD**	**vs. stable ASD**	**vs. lost diagnosis**
ADOS CSS						
Time 1	0.92 (0.45)^a^	2.13 (0.45)^c^	4.29 (0.49)^c^	−3.37 (0.62)^c^	−2.15 (0.62)^c^	−1.22 (0.59)^a^
Time 2	4.25 (0.45)^c^	3.16 (0.45)^c^	5.05 (0.49)^c^	−0.8 (0.62)	−1.89 (0.62)^b^	1.09 (0.59)
Time 3	6.04 (0.45)^c^	0.61 (0.45)	5.33 (0.49)^c^	0.71 (0.62)	−4.72 (0.62)^c^	5.43 (0.59)^c^
Non-verbal IQ						
Time 1	−3.69 (3.23)	−12.34 (3.23)^c^	−15.12 (3.52)^c^	11.42 (4.47)^a^	2.77 (4.47)	8.65 (4.25)^a^
Time 2	−25.31 (3.23)^c^	−15.4 (3.23)^c^	−26.37 (3.59)^c^	1.06 (4.53)	10.97 (4.53)^a^	−9.91 (4.25)^a^
Time 3	−14.49 (4.03)^c^	−7.59 (3.51)^a^	−33.87 (4.18)^c^	19.37 (5.42)^c^	26.28 (5.05)^c^	−6.91 (4.92)
Verbal IQ						
Time 1	−11.00 (3.88)^b^	−15.47 (3.88)^c^	−36.97 (4.22)^c^	25.97 (5.36)^c^	21.5 (5.36)^c^	4.47 (5.1)
Time 2	−28.88 (3.89)^c^	−9.76 (3.89)^a^	−31.62 (4.31)^c^	2.74 (5.43)	21.87 (5.43)^c^	−19.12 (5.1)^c^
Time 3	−24.45 (4.92)^c^	−6.54 (4.2)	−32.7 (5.13)^c^	8.25 (6.67)	26.16 (6.15)^c^	−17.91 (5.98)^b^

*SE, Standard Error; ADOS, Autism Diagnostic Observation Schedule; CSS, Calibrated Severity Score; IQ, intelligence quotient; ASD, autism spectrum disorder. Estimates are derived using linear contrasts from linear mixed-effects models with fixed effects for outcome group (Stable ASD, Later Diagnosed, Lost Diagnosis, Stable non-ASD) and time point (Time 1, Time 2, Time 3) and interactions between group and time point, including random effects for child-specific intercept*.

a *p < 0.05*.

b *p < 0.01*.

c *p < 0.001*.

Compared to the Stable ASD group, the Later Diagnosed group showed significantly lower ADOS CSS, and higher verbal and non-verbal IQ at Time 1; however, over time, non-significant group differences were observed in ADOS CSS and verbal IQ. While no group differences between the Later Diagnosed and Stable ASD groups were observed at Time 2, the Later Diagnosed group showed significantly higher non-verbal IQ than the Stable ASD group at Time 3.

Compared to the Stable ASD group, the Lost Diagnosis group showed significantly lower ADOS CSS, and higher verbal and non-verbal IQ across all time points, except for Time 1, where non-significant group differences were observed in non-verbal IQ.

Finally, compared to the Lost Diagnosis group, the Later Diagnosed group showed significantly lower ADOS CSS at Time 1, non-significant differences in ADOS CSS at Time 2, and significantly higher ADOS CSS at Time 3. In terms of non-verbal IQ, at Time 1 the Later Diagnosed group showed significantly higher non-verbal IQ; however, at Time 2 exhibited significantly lower non-verbal IQ, with non-significant group differences observed at Time 3. In terms of verbal IQ, while non-significant group differences were observed at Time 1, the Later Diagnosed group showed significantly lower verbal IQ at Times 2 and 3.

### Early Predictors of Diagnostically Stable and Dynamic Groups

At Time 1, greater limitations in IJA significantly predicted the likelihood of belonging to the Later Diagnosed and Lost Diagnosis groups, compared to the Stable non-ASD group, but not the Stable ASD group. Lower expressive language was predictive of belonging to the Later Diagnosed and Stable ASD groups, but not the Lost Diagnosis group (see Table 6). RRB was predictive of belonging to the Later Diagnosed and Stable ASD groups, but not the Lost Diagnosis group.

**Table 6:** Parameter estimates of the variables in the multinomial logistic regression model, in which the variables with *p*-values indicated in bold text, significantly predict the likelihood of belonging to the indicated 5.7 year outcome group.

**Time**	**Group**	**Predictor variable**	**Coefficient**	**SE**	***z*-value**	***p*-value**
1	Later diagnosed	**MSEL EL**	**−0.14**	**0.04**	**−3.73**	**<0.001**
		**ADOS IJA**	**0.49**	**0.17**	**2.93**	**0.003**
		**ADOS RRB**	**0.49**	**0.16**	**3.04**	**0.002**
	Lost diagnosis	MSEL EL	−0.03	0.02	−1.44	0.15
		**ADOS IJA**	**0.25**	**0.12**	**2.09**	**0.04**
		ADOS RRB	0.20	0.15	1.38	0.17
	Stable ASD	**MSEL EL**	**−0.06**	**0.02**	**−2.79**	**0.01**
		ADOS IJA	0.06	0.11	0.52	0.60
		**ADOS RRB**	**0.32**	**0.14**	**2.24**	**0.02**
2	Later diagnosed	**MSEL EL**	**−0.10**	**0.03**	**−3.52**	**<0.001**
		**ADOS IJA**	**0.55**	**0.21**	**2.58**	**0.010**
		**ADOS RRB**	**0.95**	**0.22**	**4.35**	**<0.001**
	Lost diagnosis	**MSEL EL**	**−0.09**	**0.03**	**−3.33**	**0.001**
		**ADOS IJA**	**0.39**	**0.18**	**2.15**	**0.03**
		**ADOS RRB**	**0.98**	**0.20**	**4.88**	**<0.001**
	Stable ASD	**MSEL EL**	**−0.05**	**0.03**	**−2.04**	**0.04**
		ADOS IJA	0.26	0.15	1.74	0.08
		**ADOS RRB**	**0.59**	**0.16**	**3.64**	**<0.001**

*SE, Standard Error; MSEL, Mullen Scales of Early Learning; EL, expressive language; ADOS, Autism Diagnostic Observation Schedule; IJA, initiation of joint attention ADOS composite; RRB, Restricted and Repetitive Behaviors ADOS domain score*.

At Time 2, all three predictor variables (greater limitations in IJA, lower expressive language skills, and higher levels of RRB) significantly predicted the likelihood of being in one of the ASD diagnosis groups (Later Diagnosed, Lost Diagnosis, Stable ASD) compared to the Stable non-ASD group (Table 6) *except* IJA in the Stable ASD group.

## Discussion

We examined ASD diagnostic stability beginning at the end of the ASD prodromal period, with follow-up assessments at the age of three years (when ASD diagnosis is considered to yield high stability), and at early school-age, when contextual demands on social and language processing increase substantially and could exceed children's compensatory abilities, thereby permitting first ASD detection. Our sample consisted of younger siblings of children with ASD, who are at elevated likelihood of developing ASD (ELA) (28). Further, we examined whether groups, defined by their ASD diagnostic status over the three time points, differed in trajectories of autism symptom severity, and verbal IQ and non-verbal IQ. Finally, predictors of stability groupings were examined. The present study is the largest to date to examine ASD diagnostic stability in ELA children into early school-age, and the first to examine stability in ELA children beginning at mean age 15 months (7, 12). Our findings that a meaningful proportion of ELA children shift into or out of ASD diagnosis demonstrate the non-linearity of ASD diagnostic status and the dynamic process of development. These findings also highlight the importance of ongoing autism screening beyond the preschool years, and of considering children's phenotypic presentation across development as a dynamic phenomenon.

Using diagnostic status (ASD vs. non-ASD) at school-age as the standard, we found that stability of ASD improved from Time 1 (mean age 15.4 months) to Time 2 (mean age 36.6 months), but overall, was modest (PPV: 63% and 68%, respectively). The dynamic nature of ASD symptom expression over time was evident in our sample. From mean age 15.4 months to mean age 5.7 years, 46 of the 65 (70.7%) children who received an ASD diagnosis at one or more time points shifted to or from ASD diagnosis across one or more time points. However, between Times 2 and 3, such shifting narrowed to 23 of 59 (39%) children diagnosed with ASD at either or both age(s). In Ozonoff et al. (7) and Zwaigenbaum et al. (12), such shifts occurred between 18, 24, and 36 months in 69.6 and 84.7% of ELA children, respectively. In Shephard et al. (30) and Brian et al. (29), such shifts were observed between 3 and 7 years in 53.3% of ELA children, and between 3 and 9.5 years in 29.2% of ELA children, respectively. Thus, this dynamic phenomenon is evident across studies and narrows with increasing age.

When considering reports of ASD diagnostic stability, numerous factors must be taken into account. Stability (PPV) is likely to be highest when diagnosis is made on a pre-screened sample and over a short timeframe. This has been demonstrated in a community sample that screened positive for ASD (26), as well as in a clinic sample referred to rule out ASD (1). Furthermore, stability of ASD diagnosis is likely optimized when diagnosis is made by experts whose primary focus is on early detection of ASD, particularly in federally-funded research programs where rigor and systematicity of the diagnostic process are high (e.g., calibration amongst diagnosticians, use of state-of-the-science diagnostic procedures). Yet even in such ideal diagnostic contexts, shifts in ASD diagnostic classification occur.

Between 18 and 36 months of age, the greatest proportion of instability of diagnostic classification appears to be related to autism symptom consolidation across the second year of life, with children shifting from non-ASD to ASD (7, 12). Of note, our finding of high stability of “non-ASD” classification (NPV) is consistent with other stability studies focused on ELA samples, regardless of whether diagnostic “outcome” was established at age 3, 7, or 9.5 years (7, 12, 29, 30).

In prior ASD stability studies, children demonstrating a shift from non-ASD to ASD are referred to as later diagnosed, or false negatives. The number of children receiving a later diagnosis of ASD at Time 3 in the present study dropped from 20 to 6 from Time 1 to Time 2, similar to the considerable decrease in Later Diagnosed from 18 to 24 months in previous reports (7, 12). Eighty-five percent of Later Diagnosed cases in the present study were diagnosed by age 3 years, indicating consolidation of ASD symptomatology by the third birthday.

Taking a dimensional approach, we observed that the Later Diagnosed group exhibited non-verbal cognitive functioning similar to that of the Stable non-ASD group at Time 1. However, the Later Diagnosed group increasingly departed from typical functioning, with non-verbal IQ dropping by over one standard deviation between Times 1 and 2. The slight rebound between Times 2 and 3 did not position this group to perform as well as the Stable non-ASD group on the non-verbal IQ measure at Time 3. Despite their strong non-verbal IQ performance at Time 1, the Later Diagnosed group exhibited significantly lower verbal IQ and significantly higher ASD symptoms at all time points compared to the Stable non-ASD group, with increasing differences emerging over time. In addition, only the Later Diagnosed group failed to show an upward trend in verbal IQ from Time 1 to Time 2 before stabilizing. These findings align with Landa et al. (33) showing the absence of a spurt in language development, decline in self-generated social behaviors (e.g., shared positive affect), and initial intermediate level of initiation of joint attention without the expected increase in such skills observed between 14 and 24 months in later-diagnosed children with ASD. When compared to the Stable ASD group, the Later Diagnosed group exhibited significantly higher non-verbal and verbal IQ along with lower ASD symptom severity at Time 1 and significantly higher non-verbal IQ at Time 3. No significant differences were detected between the Later Diagnosed and Stable ASD groups at Times 2 or 3 in verbal IQ or ASD symptom severity. In sum, the Later Diagnosed group was already distinguishable from the Stable non-ASD group at Time 1 yet without sufficient indicators to qualify for an ASD diagnosis. However, at the third birthday, the Later Diagnosed and Stable ASD groups were indistinguishable on the measures employed here.

Further insights into late ASD diagnosis are provided by Davidovitch et al.'s (6) study involving children diagnosed with ASD after age 6, who had received comprehensive multidisciplinary assessments at earlier ages. Data were gathered from a chart review of records in a population-based registry (6). Of the 2,543 children who received an ASD diagnosis before age 12 years, 8.7% were first diagnosed with ASD after age 6. Less than 50% had ASD characteristics noted in their medical chart. The diagnostic impressions provided by the assessing professional(s) included language impairments (70%), problems with attention (46%), and difficulties with cognitive functioning (42%). Similarly, in a detailed report of 14 children with late diagnosis of ASD from three large international prospective, longitudinal studies of children with and without ELA (32), detailed behavioral assessments at preschool-age revealed that half had demonstrated subtle, subthreshold symptomatology associated with ASD. The other half of the later-diagnosed ASD sample (*n* = 7) previously had shown neurotypical developmental profiles (32). Neurodevelopmental problems subthreshold for ASD diagnosis during childhood also were reported in adults who had late ASD diagnosis in a population-based longitudinal study (53).

The other dynamic diagnosis group identified in the present study consisted of children shifting from ASD to non-ASD classification, referred to as Lost Diagnosis. At Time 1, the Lost Diagnosis group's ASD symptom severity was comparable to that of the Stable ASD group, but their non-verbal and verbal IQ scores were significantly higher. At subsequent time points, the Lost Diagnosis group continued to display significantly higher non-verbal and verbal IQ scores but, notably, showed significantly lower ASD symptom severity compared to the Stable ASD group. The greatest drop in ASD symptom severity occurred between age 3 years and school-age.

Comparing our two dynamic diagnostic groups (Later Diagnosed and Lost Diagnosis) revealed that the Lost Diagnosis group showed significantly higher ASD symptom severity at Time 1 and significantly lower ASD symptom severity at Time 3 than the Later Diagnosed group. In the cognitive domain, at Time 1, the Lost Diagnosis group showed significantly lower non-verbal IQ than the Later Diagnosed group. At Time 2, however, the Lost Diagnosis group exhibited significantly higher non-verbal IQ than the Later Diagnosed group; the Lost Diagnosis group's verbal IQ was significantly higher than that of the Later Diagnosed group at Times 2 and 3. The type of substantial gain in verbal IQ and reduction in ASD symptom severity observed in our Lost Diagnosis group from age 15 months to school-age is phenomenologically similar to the Very Positive Outcome (VPO) group that Lord et al. (22) identified in their longitudinal study of children with ASD from age 2–19 years. The VPO group in that study, representing 10% of the sample, had verbal IQs of about 70 at two years of age, somewhat similar to that observed in our Lost Diagnosis group at age 15 months. By age 9 years, the VPO group had achieved average verbal IQ scores and nearly typical social functioning (22). In a study of different children with ASD from age 2 to 14 years, Fountain et al. (54) reported that about 10% of children with severe ASD had rapid improvement in cognitive functioning accompanied by a reduction in autism symptomatology. Together, these studies demonstrate that ASD is not a fixed diagnosis for all children.

We examined the significance of three possible Time 1 and 2 predictors (IJA, expressive language, RRB) of Time 3 membership in the three groups having received an ASD diagnosis at one or more of the three assessments points, using the Stable non-ASD group as the reference. Predictive value of these three behavioral dimensions differed for these groups and by Time. Interestingly, at Times 1 and 2, low levels of expressive language and IJA along with elevated RRB predicted Later Diagnosed group membership. This highlights the importance of even subtle variations in these domains during early development, especially in ELA children. Membership in the Lost Diagnosis group was predicted at Time 1 only by a low level of IJA. Greater expressive language skills (likely reflecting better representational and communication skills) and lower levels of RRB (possibly reflecting greater attentional and cognitive flexibility) may function as resiliency factors (47). By Time 2, predictors of membership in the Lost Diagnosis group expanded to include all three variables, which is not surprising given that 74% of the children in this group were diagnosed with ASD at Time 2. For the Stable ASD group, Time 1 and 2 predictors were identical, and included low levels of language along with elevated RRB, but not IJA. Thus, early manifestation of ASD does not necessarily mean that IJA is absent or nearly non-existent.

To understand the dynamic nature of ASD symptom severity and IQ identified in our study, and by others, it is helpful to consider ontogenetic adaptation processes (38). Through these processes, each individual's brain optimizes its fit to the environment, giving rise to behavioral phenotypes. Atypical brain characteristics associated with ASD identified in infancy likely give rise to atypical signal processing well before behavioral symptoms arise (55). Atypical signal processing likely triggers alterations in frequency and quality of engagement with the environment, generating bidirectional influences that further shape developmental trajectories (10). A cascading effect follows, where multiple developmental systems are shaped by earlier experiences. Over time, there is an accruing effect of atypical early recurrent moment-by-moment experiences on brain structures and connections as well as behavioral characteristics. Our results highlight the importance of early identification of children, at least those with a known family history of ASD, showing limited IJA, limited expressive language skills, and/or elevated RRBs and providing developmental enrichment (56, 57).

Considering our Lost Diagnosis group, and reports of improvement in children with ASD by others, properties of brain development, such as synaptic plasticity, contribute to compensatory processes. In compensatory processes, alternative routes are taken to perform activities when there is disruption in the usual routes (58). In some cases, resulting behavioral features are indistinguishable from that of neurotypical children. In other cases, compensatory processes including synaptic plasticity (59), may not achieve more developmentally advanced levels or substantially reduced ASD symptom expression due to limited resources and inefficiencies at the neuronal and neural network levels. Female sex is one possible protective factor that has been proposed in the literature (52). In support of this hypothesis, the current study found that the Lost Diagnosis group consisted of a significantly greater proportion of females vs. males, compared to the Later Diagnosed group. These results are in line with findings from Landa et al. (47) where the proportion of siblings at elevated likelihood of ASD in a “Developmental Diversity cluster” was significantly greater than in the Resiliency cluster, and the sex ratio across the two clusters differed, with proportionally more males in the Developmental Diversity Cluster relative to the Resiliency Cluster. Taken together, these finding suggest that, over time, females may have better compensatory or adaptation mechanisms relative to males. Understanding the degree to which compensatory processes are associated with neurotypical rates, degrees, and efficiencies will require highly sensitive measures of information processing using methods such as eye-tracking, electroencephalography, and functional imaging technologies.

Our findings have numerous implications for clinicians, as well as for child health advocacy and policy groups (e.g., the American Academy of Pediatrics, Centers for Disease Control), and community organizations (e.g., child care and education administrators). One implication is that screening for ASD, social, and communication functioning should not end at age 24 months (60), but should be part of health care and educational monitoring through at least middle childhood (41, 61). Furthermore, follow-up clinical evaluations of children with suspected or documented ASD should be thorough when there is evidence of phenotypic shifting, incorporating semi-structured behavioral assessments (e.g., ADOS-2), parent questionnaires (32), and examiner rating tools to detect context-dependent variations in social communication behavior (41, 61). This would permit documentation of dimensional features related to ASD that may elucidate diagnostic shifts and identification of needed changes in environmental supports. Finally, there are many children whose ASD symptoms hover at the border of diagnosable ASD. Depending on those children's other characteristics, such as IQ and adaptive functioning, clinicians may be more or less inclined to diagnose ASD. In young children, the behavioral phenotypic ‘distance' between ASD and non-ASD may be small (34). Thus, early ASD evaluations require use of measures of quantitative, dimensional clinical features to guide clinical decision making. The possibility that shifting in ASD diagnosis may occur, and indeed, that development is a dynamic process, is essential for clinicians to acknowledge, especially when supporting families' understanding of discrepancies over time in their child's diagnosis or assessment results. As developmental changes, toward or away from “typical” functioning, may happen in relatively small intervals of time, families, intervention providers, and educators must remain nimble, providing children with the type and amount of support they need to experience success and high quality of life.

Limitations of the present study include the modest sample of children with ASD (*n* = 42) at outcome, though this is at least 2.5 times larger than sample sizes in other studies of ELA children followed into school-age (29, 30). In addition, CBE diagnosis was made by one rather than two independent research clinicians at each time point. However, our research examiners were senior clinical researchers with extensive experience and expertise in early diagnosis of ASD. All examiners were trained to research reliability in ASD diagnosis and participated in ongoing, routine ADOS and diagnostic calibration meetings to monitor fidelity of diagnostic accuracy. Another factor that could be considered a limitation is the use of items derived from the ADOS to generate the IJA composite and RRB domain score in the predictor analysis. However, it is unlikely that results were significantly confounded by collinearity of these items and ADOS scores. Only two of the IJA-related items and up to four of the RRB items contribute to the ADOS algorithm, and there are many other algorithm items that contribute to ASD cut-offs. Regarding parent-report data and other clinical judgments, it was beyond the scope of this paper to report on more detailed phenotypic characteristics of the children in each of our groups. Another limitation is the lack of data available on children's early intervention and daily life experiences (e.g., screen time exposure, enrollment in childcare or other group contexts with age peers), which could have influenced ASD diagnostic stability. Strengths of this study include the large number of ELA children, inclusion of children very near to their first birthday, presentation of data obtained at three important developmental periods, examination of categorical and dimensional data across multiple aspects of development, and rigorous and expert diagnostic procedures.

Future studies examining ASD diagnostic stability should include research methods permitting examination of information processing, such as eye-tracking or electroencephalography, to better understand brain compensatory processes and their relation to the expression of ASD symptoms over time. Future studies should also include samples at elevated likelihood of developing language disorders or attention-deficit/hyperactivity disorder, with assessments beginning early in the second year of life, to elucidate additional ASD predictors, overlap of dimensional traits, compensatory processes, and trajectories associated with what have historically been considered categorically distinct developmental conditions. Lastly, much of the existing work on developmental trajectories has been performed in multiplex families, given the relative efficiency of early-life longitudinal studies in enriched elevated-likelihood populations (7, 12, 29, 30, 32–34). Recent work by Pierce et al. (26) found that, among toddlers recruited from the general community who received their first diagnostic evaluation between 12 and 36 months, 23.8% were not diagnosed as ASD until a later visit, which is a substantially lower estimate than those from infant sibling studies (50–80%) (7, 12), possibly because the children referred for assessment had screened positive for ASD. More research is needed to replicate this finding and further explore developmental trajectories in general population cohorts. Such research should help to advance the field's appreciation of developmental and neurobiological systems, as well as support clinicians' and educators' ability to focus on each child's unique learning strengths and needs and to tailor environmental supports accordingly.

## Data Availability Statement

The data that support the present study are included in the article. Further inquiries can be directed to the corresponding author.

## Ethics Statement

The studies involving human participants were reviewed and approved by Johns Hopkins University School of Medicine Institutional Review Board. Written informed consent to participate in this study was provided by the participants' legal guardian/next of kin.

## Author Contributions

RL conceived of the original study from which the present study emerged and presented the idea for the present study. RL, RR, and CBH conceived of the data analytic plan. CBH verified the analytical methods. DH and CRH conducted the child assessments and made diagnostic classifications. CBH, RR, and DH prepared the data for analysis. RR conducted the analyses. RL and RR wrote the manuscript. All authors contributed to the refinement of the research questions. All authors discussed results, critically reviewed drafts of the manuscript, and approved the final draft.

## Funding

This study was funded by the National Institute of Mental Health R01 MH59630 grant (PI: RL). We acknowledge resources provided by the Kennedy Krieger Institute Intellectual and Developmental Disabilities Research Center (P50 HD103538).

## Conflict of Interest

The authors declare that the research was conducted in the absence of any commercial or financial relationships that could be construed as a potential conflict of interest.

## Publisher's Note

All claims expressed in this article are solely those of the authors and do not necessarily represent those of their affiliated organizations, or those of the publisher, the editors and the reviewers. Any product that may be evaluated in this article, or claim that may be made by its manufacturer, is not guaranteed or endorsed by the publisher.
